# Fully automated closed-loop glucose control compared with standard insulin therapy in adults with type 2 diabetes requiring dialysis: an open-label, randomized crossover trial

**DOI:** 10.1038/s41591-021-01453-z

**Published:** 2021-08-04

**Authors:** Charlotte K. Boughton, Afroditi Tripyla, Sara Hartnell, Aideen Daly, David Herzig, Malgorzata E. Wilinska, Cecilia Czerlau, Andrew Fry, Lia Bally, Roman Hovorka

**Affiliations:** 1 Wellcome Trust – MRC Institute of Metabolic Science, Addenbrooke’s Hospital, Cambridge, UK; 2 Cambridge University Hospitals NHS Foundation Trust, Wolfson Diabetes and Endocrine Clinic, Cambridge, UK; 3 Department of Diabetes, Endocrinology, Nutritional Medicine and Metabolism, Inselspital, Bern University Hospital and University of Bern, Bern, Switzerland; 4 Department of Nephrology and Hypertension, Inselspital, Bern University Hospital and University of Bern, Bern, Switzerland; 5 Department of Renal Medicine, Cambridge University Hospitals NHS Foundation Trust, University of Cambridge, Cambridge, UK

**Keywords:** Type 2 diabetes, End-stage renal disease

## Abstract

We evaluated the safety and efficacy of fully closed-loop insulin therapy compared with standard insulin therapy in adults with type 2 diabetes requiring dialysis. In an open-label, multinational, two-center, randomized crossover trial, 26 adults with type 2 diabetes requiring dialysis (17 men, 9 women, average age 68 ± 11 years (mean ± s.d.), diabetes duration of 20 ± 10 years) underwent two 20-day periods of unrestricted living, comparing the Cambridge fully closed-loop system using faster insulin aspart (‘closed-loop’) with standard insulin therapy and a masked continuous glucose monitor (‘control’) in random order. The primary endpoint was time in target glucose range (5.6–10.0 mmol l^−1^). Thirteen participants received closed-loop first and thirteen received control therapy first. The proportion of time in target glucose range (5.6–10.0 mmol l^−1^; primary endpoint) was 52.8 ± 12.5% with closed-loop versus 37.7 ± 20.5% with control; mean difference, 15.1 percentage points (95% CI 8.0–22.2; *P* < 0.001). Mean glucose was lower with closed-loop than control (10.1 ± 1.3 versus 11.6 ± 2.8 mmol l^−1^; *P* = 0.003). Time in hypoglycemia (<3.9 mmol l^−1^) was reduced with closed-loop versus control (median (IQR) 0.1 (0.0–0.4%) versus 0.2 (0.0–0.9%); *P* = 0.040). No severe hypoglycemia events occurred during the control period, whereas one severe hypoglycemic event occurred during the closed-loop period, but not during closed-loop operation. Fully closed-loop improved glucose control and reduced hypoglycemia compared with standard insulin therapy in adult outpatients with type 2 diabetes requiring dialysis. The trial registration number is NCT04025775.

## Abstract (web summary)

A new randomized, crossover clinical trial testing rapid-acting insulin aspart delivered in a closed-loop system compared with standard care demonstrates increased time in range in patients with type 2 diabetes who require dialysis in the outpatient setting.

## Main

Diabetic nephropathy is the most common cause of end-stage renal disease (ESRD), accounting for 30% of incident cases in the UK and 29% in Europe in 2018^1,2^. As the prevalence of type 2 diabetes increases, the number of people with diabetes and ESRD requiring renal replacement therapy is also rising^1^. A scarcity of organs for transplantation as well as cardiovascular comorbidities associated with diabetes that preclude transplantation mean that hemodialysis or peritoneal dialysis are the only available treatment options for many.

ESRD and dialysis itself increase the risk of hypoglycemia and hyperglycemia, which are associated with adverse outcomes^3–5^. Management of diabetes in this population is challenging for both patients and healthcare professionals. Many aspects of diabetes care of patients on dialysis are poorly understood, including targets for glycemic control and treatment algorithms^6,7^. Most oral diabetes medications are contraindicated in people with ESRD, so insulin is the most commonly used diabetes therapy. Optimal insulin dosing regimens are difficult to establish with the altered glucose and insulin metabolism associated with ESRD and dialysis^5^, and concerns regarding hypoglycemia often result in sub-optimal glycemic control. There is an unmet need for novel approaches to the safe and effective management of diabetes for people requiring dialysis.

Closed-loop insulin delivery systems comprise a continuous glucose monitor, an insulin pump and a control algorithm that continuously and automatically modulates subcutaneous insulin delivery in response to real-time interstitial glucose concentrations^8^. Closed-loop systems are increasingly being applied to the management of type 1 diabetes. However, use of this technology in people with type 2 diabetes has been limited to the inpatient setting including those on hemodialysis^9–12^. Safety and efficacy in outpatient settings, a precursor for wider clinical acceptance, is to be determined. In the present study, we address this issue and hypothesize that fully closed-loop insulin delivery may improve glycemic control compared to standard insulin therapy without increasing the risk of hypoglycemia in people with type 2 diabetes and ESRD undergoing maintenance dialysis in the outpatient setting.

## Results

### Study participants

**Fig. 1:**
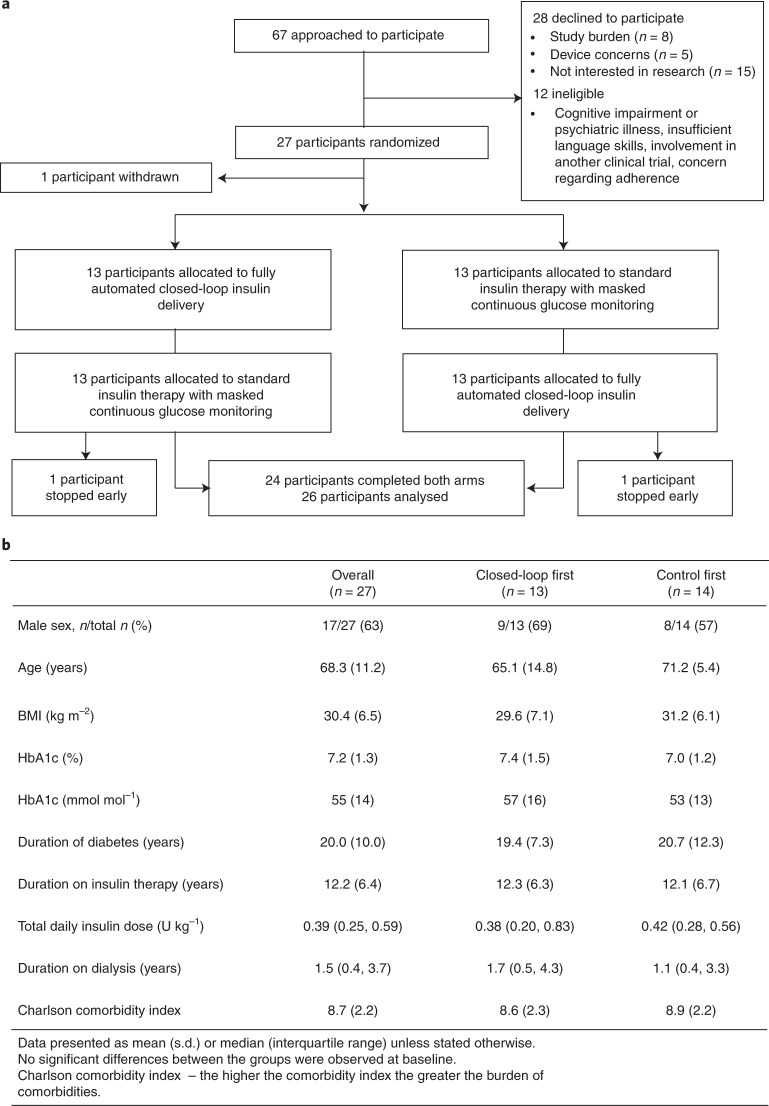
Overview of the trial and participants. **a**, Overview of the participant flow. **b**, Baseline characteristics of the study participants.

**Extended Data Fig. 1:**
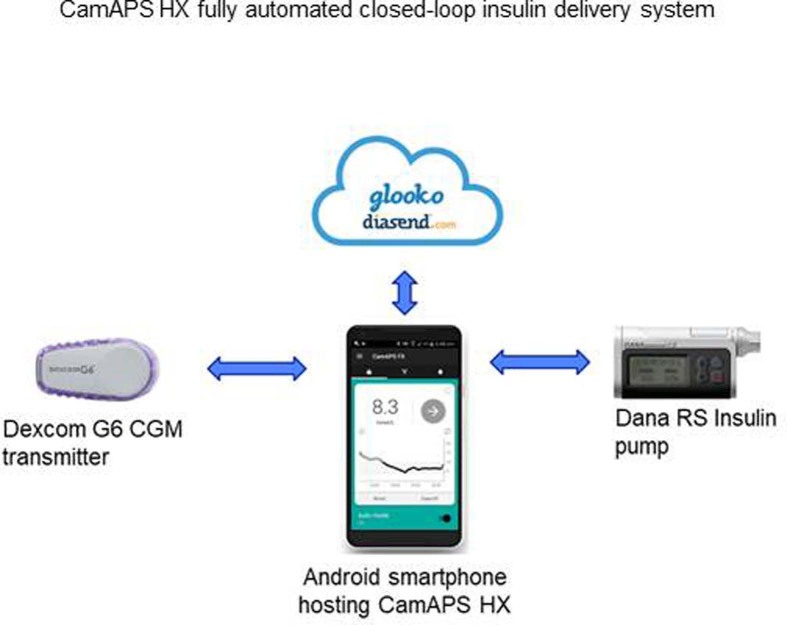
CamAPS HX fully automated closed-loop insulin delivery system. CamAPS HX fully automated closed-loop insulin delivery system.

From 21 October 2019 to 3 November 2020, 27 participants were enrolled and randomized (17 men, 9 women, average age 68 ± 11 years (mean ± s.d.) and average diabetes duration 20 ± 10 years; one participant died prior to starting the first treatment arm; Fig. 1b). Baseline diabetes regimen details are shown in Supplementary Table 1. Thirteen participants were randomized to receive closed-loop first (Extended Data Fig. 1) and thirteen were randomized to standard insulin therapy first. Recruitment stopped early due to Brexit-related sponsorship issues and delays and constraints caused by COVID-19 (Methods). The flow of participants through the trial is shown in Fig. 1a. Of 27 randomized participants, one participant was withdrawn from the study post-randomization as they required hospital admission and died before the start of the first intervention period (control). Two participants stopped a study period early, one during the second period (control) due to bereavement and one during the first period (closed-loop) due to local COVID-19 restrictions. These participants both completed a minimum of 48 h in both study periods and were included in the analysis. The average washout period was 17 ± 5 days, overall. The washout period in those receiving closed-loop first was 16 ± 4 days, and was 17 ± 5 days in those receiving usual care first.

### Efficacy

Primary and secondary endpoints calculated using data from all randomized subjects with at least 48 h of available data in both study periods (*n* = 26) are presented in Table 1. The primary endpoint, the proportion of time sensor glucose was in the target glucose range between 5.6 and 10.0 mmol l^−1^, was greater during closed-loop use than with standard insulin therapy (52.8 ± 12.5% versus 37.7 ± 20.5% for closed-loop versus control, respectively; *P* < 0.001), with a mean difference of 15.1 percentage points in favor of closed-loop (95% CI 8.0–22.2). The time in range with closed-loop in period 1 was 54.4 ± 12.1% and in period 2 was 51.2 ± 13.3%. The time in range with standard insulin therapy in period 1 was 37.1 ± 22.9% and was 38.3 ± 18.6% in period 2. No period effect was observed (*P* = 0.86).

**Table 1:** Comparison of primary and secondary outcomes between closed-loop and control periods

	Closed-loop (*n* = 26)	Control (*n* = 26)	*P* value
Proportion of time spent at glucose level (%)			
5.6–10.0 mmol l^−1 a^	52.8 (12.5)	37.7 (20.5)	<0.001
3.9–10.0 mmol l^−1^	57.1 (14.3)	42.5 (24.7)	0.002
>10.0 mmol l^−1^	42.6 (14.3)	56.6 (25.1)	0.003
>20.0 mmol l^−1^	1.8 (2.4)	6.7 (10.7)	0.012
<5.6 mmol l^−1^	3.2 (2.0, 7.0)	4.0 (0.9, 9.5)	0.87
<3.9 mmol l^−1^	0.12 (0.02, 0.44)	0.17 (0.00, 1.11)	0.040
<3.0 mmol l^−1^	0.00 (0.00, 0.03)	0.00 (0.00, 0.22)	0.047
Mean glucose (mmol l^−1^)	10.1 (1.3)	11.6 (2.8)	0.003
Standard deviation of glucose (mmol l^−1^)	3.2 (0.7)	3.6 (0.9)	0.021
CV of glucose (%)	31.7 (4.8)	31.5 (5.4)	0.87
Between days CV of glucose (%)	30.8 (3.4)	31.2 (5.8)	0.72
Total daily insulin dose (U kg^−1^)	0.34 (0.15, 0.54)	0.36 (0.19, 0.58)	0.37
Total daily insulin dose (U)	20.4 (9.2, 50.3)	32.2 (12.1, 54.4)	0.38
Sensor glucose data (h)	454 (450, 460)	452 (425, 454)	0.062
Time using sensor glucose (%)	95 (94, 96)	94 (90, 95)	0.062
Time using closed-loop (%)	93 (89, 94)	–	–

^a^Primary endpoint.

Data presented as mean (s.d.) or median (interquartile range).

CV, coefficient of variation.

A two-sample *t*-test on paired differences was used to compare normally distributed variables and the Mann–Whitney–Wilcoxon rank-sum test was used for data that are not normally distributed. No allowance was made for multiplicity.

**Fig. 2:**
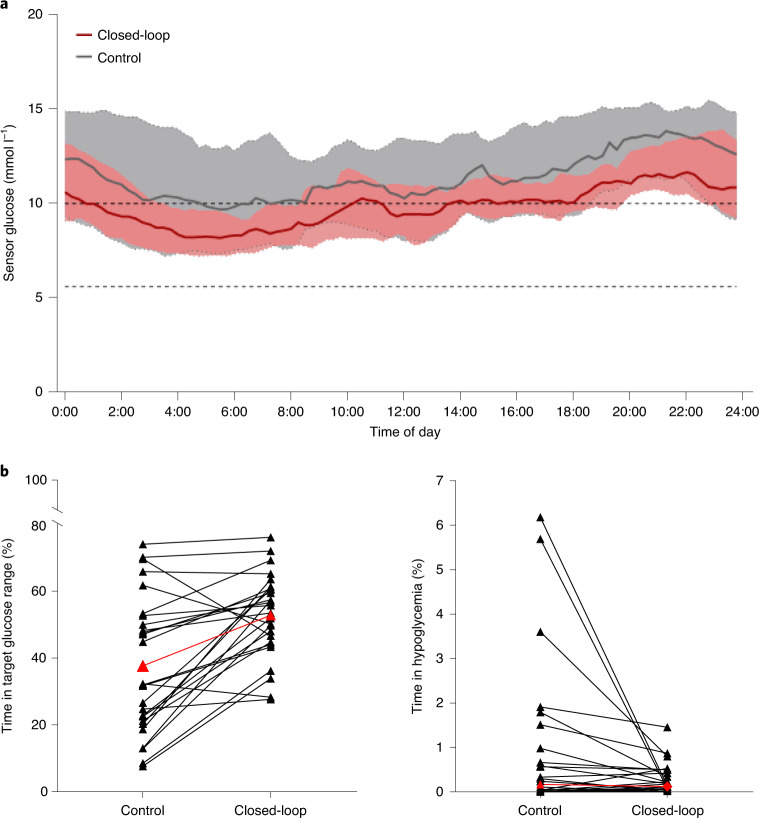
Glycemic outcomes during closed-loop and control periods. **a**, Median and IQR of sensor glucose during the closed-loop period (solid red line and pink shaded area) and control period (solid gray line and gray shaded area) from midnight to midnight. *n* = 26 biologically independent samples. The lower and upper limits of the glucose target range, 5.6–10.0 mmol l^−1^, are denoted by the horizontal dashed lines. **b**, Individual participants’ time spent with glucose in the target glucose range of 5.6–10.0 mmol l^−1^ (left; overall mean shown in red) and with glucose in hypoglycemia <3.9 mmol l^−1^ (right; overall median shown in red) during control and closed-loop therapy.

Mean glucose was lower with closed-loop than control (10.1 ± 1.3 versus 11.6 ± 2.8 mmol l^−1^ respectively; mean difference of 1.5 mmol l^−1^ (95% CI 0.6–2.5); *P* = 0.003). Figure 2 shows the 24-h sensor glucose profiles. The time spent in hypoglycemia (sensor glucose <3.9 mmol l^−1^) was reduced with closed-loop versus control (median (IQR) 0.12 (0.02–0.44%) versus 0.17 (0.00–1.11%); *P* = 0.040; Fig. 2b).

The standard deviation of glucose was lower during closed-loop than during the control period (3.2 ± 0.7 versus 3.6 ± 0.9 mmol l^−1^; *P* = 0.021) but there was no significant difference in the within-day or between-day coefficient of variation of glucose between interventions (Table 1). Total daily insulin doses were similar between interventions.

**Fig. 3:**
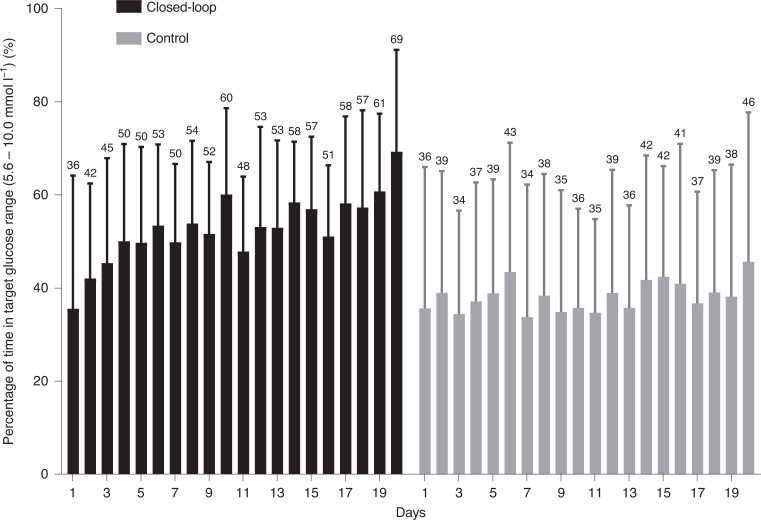
Daily trend of the proportion of time when sensor glucose was in the target range for the two treatments. Daily trend of the proportion of time when sensor glucose was in the target range between 5.6 and 10.0 mmol l^−1^ during the closed-loop period (black bars) and the control period (gray shaded bars). *n* = 26 biologically independent samples. Mean and s.d. are shown.

Closed-loop performance improved from days 1–7 to days 8–20, as shown by an increase in the time spent in the target glucose range by 8.1 percentage points (47.6 ± 16.1 versus 55.8 ± 12.6 mmol l^−1^; Fig. 3). Mean glucose and time in hyperglycemia (sensor glucose >10 mmol l^−1^) both decreased during days 8–20 without any difference in time spent in hypoglycemia or total daily insulin dose (Supplementary Table 2). There was no difference in key glycemic outcomes between days 1–7 and days 8–20 during the control period, but measures of glycemic variability increased during days 8–20 compared with days 1–7 inclusive (Supplementary Table 2).

There were no differences in any glycemic outcomes, including measures of variability between dialysis days and non-dialysis days during either intervention period (Table 2). Closed-loop driven insulin delivery was lower on dialysis days than on non-dialysis days (0.29 (0.13, 0.51) versus 0.31 (0.16, 0.53) U kg^−1^, respectively; Table 2). There was no difference in the mean inter-dialytic weight gain between interventions (closed-loop 1.8 ± 1.2 versus control 1.7 ± 1.1 kg; *P* = 0.55).

**Table 2:** Dialysis day and non-dialysis day outcomes during closed-loop and control periods

	Dialysis days		Non-dialysis days	
	Closed-loop (*n* = 25^a^)	Control (*n* = 25^a^)	Closed-loop (*n* = 25^a^)	Control (*n* = 25^a^)
Time spent at glucose levels (%)				
5.6–10.0 mmol l^−1^	53.9 (14.7)	37.2 (20.3)	51.9 (12.5)	36.3 (22.2)
>10.0 mmol l^−1^	41.0 (16.2)	56.2 (24.9)	43.5 (14.4)	58.8 (26.5)
<3.9 mmol l^−1^	0.1 (0.0, 0.3)	0.1 (0.0, 0.9)	0.0 (0.0, 0.3)	0.1 (0.0, 1.1)
Mean glucose (mmol l^−1^)	10.1 (1.5)	11.5 (2.6)	10.1 (1.3)	11.9 (3.2)
Standard deviation of glucose (mmol l^−1^)	2.8 (0.7)	3.2 (0.9)	2.8 (0.6)	3.0 (0.8)
CV of glucose (%)	27.4 (4.7)	27.8 (5.6)	28.0 (3.1)	26.1 (6.9)
Total daily insulin dose (U kg^−1^)	0.29 (0.13, 0.51)	0.36 (0.18, 0.57)	0.31 (0.16, 0.53)	0.37 (0.19, 0.59)

^a^*n* = 25, as one participant receiving peritoneal dialysis was excluded from this analysis.

Data presented as mean (s.d.) or median (interquartile range).

CV, coefficient of variation.

**Extended Data Fig. 2:**
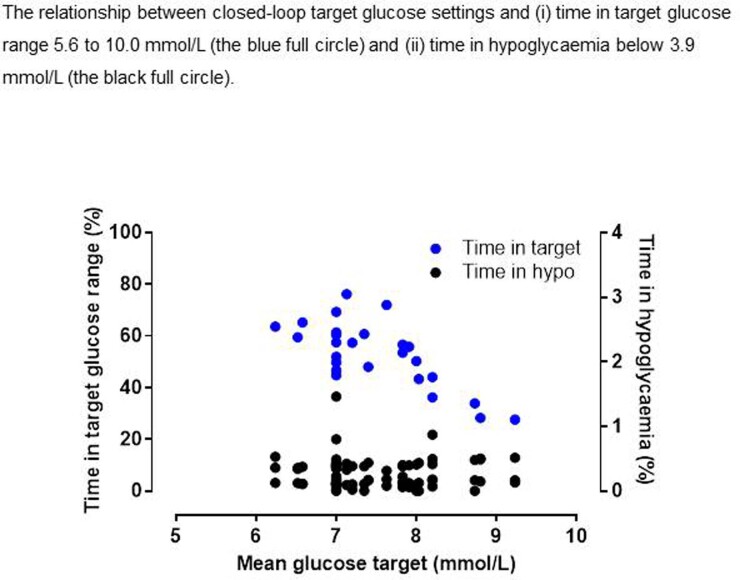
The relationship between closed-loop target glucose settings and glucose outcomes. The relationship between closed-loop target glucose settings and (i) time in target glucose range 5.6 to 10.0 mmol/L (the blue full circle) and (ii) time in hypoglycaemia below 3.9 mmol/L (the black full circle).

The closed-loop algorithm glucose target was set at 7.3 (7.0, 8.0) mmol l^−1^. The proportion of time spent in the target glucose range decreased as the glucose target setting increased (Extended Data Fig. 2).

### Safety

One episode of severe hypoglycemia occurred during the closed-loop period, but closed-loop had not been in operation at the time of the event or for 24 h previously. Six other serious adverse events were reported (Table 3). Two of these occurred during the closed-loop period (reduced responsiveness on dialysis requiring hospital admission and COVID-19 infection requiring hospital admission), two events occurred during washout or pre-study start (one hospital admission for bowel obstruction resulting in death and one hospital admission for diabetic foot-related cellulitis requiring intravenous antibiotics), and two events occurred during the control period (one below-knee amputation due to diabetic foot ulceration, and one hospital admission with an ischemic stroke). None of the serious adverse events were deemed related to study devices or study procedures.

**Table 3:** Adverse events and safety analyses

	Overall (*n* = 27)	Closed-loop (*n* = 26)	Control (*n* = 26)
Number of severe hypoglycemia events	1	1	0
Number (%) of participants with severe hypoglycemic events	1 (4)	1 (4)	0 (0)
Number of serious adverse events (not study-related)	7	3	2
Number (%) of participants with serious adverse events	6 (22)	3 (12)	2 (8)
Number of other adverse events	9	5	2
Number (%) of participants with adverse events	7 (26)	4 (15)	2 (8)
Number of device deficiencies	6	5	1
Number (%) of participants with device deficiencies	6 (22)	5 (19)	1 (4)

Severe hypoglycemia is defined as capillary glucose < 2.2 mmol l^−1^ or requiring assistance of another person.

Nine other adverse events were reported (Table 3), five of which occurred during closed-loop, two during the control period and two during washout or pre-study arm start. Three of these events were deemed related to study devices or study procedures (two skin reactions from the infusion sets and one infusion set failure causing hyperglycemia). Six device deficiencies occurred during the entire study (three sensor-related, one phone/receiver-related and two closed-loop initiation errors), none of which led to an adverse event.

### Utility evaluation and diabetes burden

Glucose sensor and closed-loop usage were high in the study, at 95% (94, 96) and 93% (89, 94), respectively. The hypoglycemia confidence score was higher with the closed-loop system than with standard insulin therapy (3.8 versus 3.5, *P* = 0.013), but there was no difference between interventions in the hypoglycemia worry score or diabetes burden measured by the ‘problem areas in diabetes’ (PAID) survey (Supplementary Table 3). The PAID score in both periods of the study was low (7.5 for control, 10.0 for closed-loop; highest score of 100.0).

All responders (*n* = 24) reported that they were happy to have their glucose levels controlled automatically by the closed-loop system and would recommend the closed-loop system to others. Ninety-two percent (*n* = 22) reported that they spent less time managing their diabetes with the closed-loop system than in the control period, and 87% (*n* = 21) were less worried about their glucose levels with the closed-loop system than with standard insulin therapy (Supplementary Table 4). Fifty percent (*n* = 12) of responders reported improved sleep and 8% (*n* = 2) reported worse sleep while using closed-loop.

Benefits of the closed-loop system reported by study participants included a reduced need for finger-prick glucose checks, less time required to manage diabetes, resulting in more personal time and freedom, and improved peace of mind and reassurance. Device burden and discomfort wearing the insulin pump and carrying the smartphone were the most common limitations reported by participants (Supplementary Table 4).

## Discussion

This study provides evidence that fully closed-loop insulin delivery can improve glucose control and reduce hypoglycemia compared to standard insulin therapy in adults with type 2 diabetes and ESRD requiring dialysis, in an unrestricted home setting. We have shown that the fully closed-loop system has the potential to safely and effectively manage glucose levels in one of the most vulnerable subpopulations with type 2 diabetes where the risk of glycemic complications and diabetes-related adverse events is greatest.

Compared with control therapy, fully closed-loop insulin delivery was associated with over 3.5 additional hours every day spent in the target glucose range. The efficacy of closed-loop directed insulin delivery improved considerably over the study period with algorithm adaptation, and time in the target glucose range increased from 36% on day 1 to over 60% by the end of the 20-day intervention period (Fig. 3). This finding highlights the importance of an adaptive algorithm that can adjust in response to individuals’ changing insulin requirements over time, independent of its initialization. This pattern of incremental improvements in time in range with increasing duration of wear time has been reported previously with this fully closed-loop system in the inpatient setting^10^. It is reasonable to postulate that time in target range could improve further with a longer duration of use. It has previously been reported that 26 days of closed-loop use are required for the proportion of time in target glucose range to plateau, although this is likely to be population-dependent^13,14^.

In this study, the proportion of time in target range with closed-loop was lower than observed in a retrospective analysis of inpatients requiring hemodialysis using the same algorithm in a hospital setting (53% versus 69%, respectively)^12^. A higher glucose target was applied in the present study (median 7.3 mmol l^−1^ versus 5.8 mmol l^−1^), given the vulnerable population, which probably contributed to the reduced time in target glucose range observed. Higher glucose target settings were associated with less time in target glucose range (Extended Data Fig. 2). However, time spent in hypoglycemia did not increase with lower personal glucose targets, suggesting that the glucose target does not need to be unnecessarily elevated.

The reduction in time in hypoglycemia observed with closed-loop is clinically important in this highly vulnerable population with a high burden of comorbidities. Closed-loop was associated with very low time in hypoglycemia (0.12% time spent with glucose <3.9 mmol l^−1^), despite accommodating the glycemic excursions associated with end-stage renal failure and dialysis. Hypoglycemia exposure during the control period was also low, in contrast with the high frequency of hypoglycemia reported in other studies^15,16^. The greatest reductions in hypoglycemia with closed-loop were observed in participants with the highest levels of hypoglycemia during the standard insulin therapy period (Fig. 2b). Hypoglycemia is a considerable barrier to optimization of insulin therapy. The risk of hypoglycemia is high in this population, and people on dialysis often have impaired awareness of hypoglycemia^17^. Hypoglycemia has been associated with an increased risk of all-cause mortality in those with diabetes on dialysis, but causation has not been established^17^.

The improved time in target glucose range observed with closed-loop was predominantly due to the reduced time spent in hyperglycemia. Time spent with glucose levels in severe hyperglycemia (>20.0 mmol l^−1^) was also reduced with closed-loop therapy. This degree of hyperglycemia is associated with both acute and chronic complications.

The closed-loop algorithm was able to manage fluctuations in glucose and insulin kinetics between dialysis and non-dialysis days effectively. There was no difference in glucose outcomes between dialysis and non-dialysis days, but closed-loop insulin delivery was lower on dialysis days than non-dialysis days, an effect that is probably related to the impact of the dialysate glucose concentration on blood glucose concentrations.

Closed-loop insulin delivery was safe in this vulnerable population. Although there was one severe hypoglycemia episode during the closed-loop arm, this occurred when closed-loop had not been in operation for over 24 h. No study-related serious adverse events occurred during the closed-loop intervention period, and the commonest study-related adverse events were self-limiting skin reactions.

Closed-loop and sensor glucose usage were high in the study, supporting acceptability of this approach in this population. All study participants were happy to have glucose levels managed with an automated insulin delivery system and would recommend its use to others. Participants felt more confident in managing hypoglycemia with the closed-loop system, although this could be due to the availability of real-time glucose levels and alarms for hypoglycemia. Device burden was reported as the main perceived drawback to this approach.

The strengths of this study include the multinational randomized crossover design, the fully closed-loop approach adopted and the unrestricted and unsupervised home setting, including dialysis sessions.

Limitations include the smaller sample size than planned due to Brexit-related study sponsorship issues and the COVID-19 pandemic. Device management was performed by the study team to minimize training burden and therefore we cannot comment on the competency of this population to self-manage this treatment modality. Diabetes therapies during the control period were not standardized or optimized during the trial. We did not evaluate the accuracy of the glucose sensor in the present study; however, because the same sensor was used during both study arms, we believe this is unlikely to have impacted the results. As this was an exploratory study, no adjustment was made for multiple comparisons in the statistical analysis. We included only one participant receiving peritoneal dialysis, thus limiting interpretation of efficacy and safety in this specific cohort.

Our study evaluated the performance of a fully closed-loop system in an unrestricted outpatient setting in a highly vulnerable population with type 2 diabetes and end-stage renal failure requiring dialysis. Having demonstrated safety and efficacy in this at-risk population in this exploratory study, larger studies are now required to confirm these findings and to determine if the glycemic improvements observed with closed-loop are associated with a reduction in complications and improved quality of life, as well as whether closed-loop should be targeted towards specific subpopulations (for example, those with high hypoglycemic burden or peri-transplant). We suggest that the fully closed-loop approach may also be beneficial in the wider population of people with type 2 diabetes, and further studies are warranted.

## Methods

### Trial design and participants

The trial adopted an open-label, two-center, multinational, randomized, two-period crossover design contrasting fully closed-loop glucose control using faster-acting insulin aspart (Fiasp, Novo Nordisk) (‘closed-loop’) and standard multiple daily insulin injection therapy (‘control’) during unrestricted living. Each intervention period lasted 20 days, separated by two to four weeks of washout using pre-study treatment. The order of the two interventions was random.

Participants were recruited from dialysis centers and nephrology and diabetes outpatient clinics at Addenbrooke’s Hospital in Cambridge, United Kingdom, and Inselspital, University Hospital of Bern, Switzerland. Inclusion criteria included age 18 years and older, type 2 diabetes requiring subcutaneous insulin therapy and end-stage renal failure requiring maintenance dialysis (hemodialysis or peritoneal dialysis). Exclusion criteria included type 1 diabetes, pregnancy or breast-feeding, severe visual or hearing impairment and any physical or psychological disease, or the use of medication(s) likely to interfere with the conduct of the trial or interpretation of the results.

Written informed consent was obtained from all participants prior to the start of study-related procedures. The study protocol was approved by the local research ethics committees (London–Stanmore Ethics Committee, UK; Ethics Committee Bern, Switzerland) and regulatory authorities (MHRA and Swissmedic). The full trial protocol is available in the Supplementary Note. The safety aspects of the trial were overseen by an independent Data and Safety Monitoring Board. The study was registered 19 July 2019 with ClinicalTrials.gov NCT04025775.

### Protocol adherence

There were 25 protocol deviations during the study period, including seven COVID-19-related deviations (delay to starting or premature finishing of a study period), seven home visits to replenish insulin supplies and 11 visits to replace infusion sets, sensors or batteries.

Recruitment was stopped early due to Brexit-related sponsorship issues that prevented the Switzerland site from recruiting any further participants after 31 December 2020, and UK study personnel were working clinically in high-risk COVID-19 environments that could have put study participants at increased risk.

### Randomization and masking

Eligible participants were randomly assigned to either initial use of fully closed-loop glucose control with faster-acting insulin aspart for 20 days followed by standard multiple daily insulin injection therapy for 20 days, or vice versa. Randomization was done using a computer-generated sequence with a permuted block design (block size 4) and stratified by center. Participants and investigators were not masked to the intervention being used during each period due to the nature of the interventions precluding the ability to mask.

### Procedures

Participant demographics and medical history, body weight and height, glycated hemoglobin (HbA1c) and total daily insulin dose were recorded at enrollment.

Body weight pre- and post-dialysis was recorded at each dialysis session (or daily if on peritoneal dialysis) as per usual clinical practice. All participants dialyzed with 5.5 mmol l^−1^ glucose-containing dialysate. Fingerstick capillary glucose measurements were performed by dialysis staff according to usual clinical practice.

### Closed-loop insulin delivery system

The CamAPS HX closed-loop app (CamDiab) resides on an unlocked Android phone, receives sensor glucose data from a Dexcom G6 transmitter (Dexcom) and uses the Cambridge adaptive model predictive control algorithm (version 0.3.71) to direct insulin delivery on a Dana Diabecare RS pump (Diabecare; Extended Data Fig. 1). Every 8 to 12 min, and based on sensor glucose data, the Cambridge adaptive control algorithm calculates an insulin infusion rate that is communicated wirelessly to the insulin pump. Sensor glucose and insulin data are automatically uploaded to the Diasend/Glooko (https://diasend.com//en) data management platform.

The control algorithm is initialized using the participant’s weight and total daily insulin dose and gradually adapts its insulin dosing based on observed glucose patterns. The nominal glucose target is 5.8 mmol l^−1^ and can be adjusted as required between 4.4 and 11.0 mmol l^−1^. In the present study, given the vulnerable population, the glucose target was set at 7.0 mmol l^−1^ and above, based on individual circumstances. Low glucose alarms were customized at a threshold to suit the participant.

### Closed-loop period

Participants’ usual insulin therapy was discontinued on the day of closed-loop initialization. All other medications were continued.

Closed-loop insulin delivery was continued for 20 days, including during dialysis sessions. Faster-acting insulin aspart (Fiasp) was delivered via the insulin pump throughout the closed-loop study period. Fiasp was used for its properties of faster onset and offset of action, and its potential to enhance closed-loop performance. No prandial insulin boluses were delivered and the control algorithm was not aware of timing or carbohydrate content of meals. Infusion sets were changed at each dialysis session by the study team.

Participants were unrestricted in relation to their usual activity and dietary intake. The study did not interfere with or specify the medications prescribed by the local clinical team. All participants were provided with a 24-h telephone helpline to contact the local study team in the event of study-related issues. At the end of the closed-loop period, devices were removed and participants’ usual insulin therapy re-started.

### Standard insulin therapy period

During the control period, participants’ received their usual insulin therapy and other diabetes medications. Fingerstick capillary glucose measurements were performed by participants as per usual clinical practice. Glycemic management was performed by the clinical team according to local practice. A continuous glucose sensor, Dexcom G6 (Dexcom), was inserted by the study team on the first day of the study arm. The continuous glucose monitor receiver was modified to mask the sensor glucose concentration to the participant and investigators.

Participants were unrestricted in relation to their usual activity and dietary intake. The study did not interfere with or specify the medications prescribed by the local clinical team. All participants were provided with a 24-h telephone helpline to contact the local study team in the event of study-related issues. At the end of the standard insulin therapy period, the glucose sensor was removed.

### Questionnaires

Participants were invited to complete the validated questionnaires at the end of each study period: the PAID questionnaire to assess diabetes distress, the Hypoglycaemia Confidence Survey to evaluate perceptions of ability to self-manage hypoglycemia and the Hypoglycaemia Fear Survey-II Worry Scale HFS-W to estimate hypoglycemia-related fear and anxiety (Cambridge only)^18–20^. Additionally, participants filled in a closed-loop experience questionnaire collecting feedback on satisfaction with closed-loop therapy, acceptance of wearing study devices and recommending closed-loop to others.

### Sample size

This was an exploratory study aiming for 32 subjects with at least 48 h of data. Because previous studies using closed-loop in an inpatient setting may not provide reliable information about the standard deviation of the primary endpoint in this particular population (outpatients receiving maintenance dialysis), no formal power calculation was applied. The sample size corresponds to the sample size of previous feasibility closed-loop randomized trials^9,11^.

### Study endpoints

The primary endpoint was the percentage of time the sensor glucose measurement was in the target glucose range of 5.6–10.0 mmol l^−1^ during the 20-day study period. This target glucose range was selected in line with recommendations for less stringent glucose control in this population due to their high risk for hypoglycemia and related adverse events^5,6,21–23^.

Other key endpoints are the percentage of time spent with sensor glucose above 10.0 mmol l^−1^, mean sensor glucose and the percentage of time spent with sensor glucose below 3.9 mmol l^−1^. Secondary efficacy endpoints included time spent with sensor glucose below 5.6 mmol l^−1^ and below 3.0 mmol l^−1^, time spent with sensor glucose levels in severe hyperglycemia (>20 mmol l^−1^) and the total daily insulin dose. Glucose variability was evaluated by the standard deviation and the coefficient of variation of sensor glucose utilizing data collected from the whole study period. The between-day coefficient of variation of sensor glucose was calculated from daily mean glucose values (0:00–23:59).

Safety endpoints included severe hypoglycemia (capillary glucose <2.2 mmol l^−1^ or requiring assistance of another person), along with other adverse events and serious adverse events and device deficiencies.

Exploratory analyses included a subset of glucose and insulin metrics during the first seven days and during the subsequent period of day 8 to day 20 (time in target, time above target, time in hypoglycemia (<3.9 mmol l^−1^), mean sensor glucose, standard deviation and the coefficient of variation of sensor glucose, and total daily insulin dose) to limit the number of comparisons. Variability of glucose and insulin requirements between dialysis and non-dialysis days was assessed using the coefficient of variation of sensor glucose and insulin requirements between dialysis days (0:00–23:59) and non-dialysis days (0:00–23:59). Mean inter-dialytic weight gain was calculated for each study period.

Psychosocial assessments were measured using questionnaires collected at the end of each study period and closed-loop participants’ experience at the end of the closed-loop period.

### Statistical analysis

The statistical analysis plan was agreed by the investigators in advance. All analyses were carried out on an intention-to-treat basis. We analyzed endpoints from participants with at least 48 h of sensor glucose data in both study periods. The respective values obtained during the 20-day randomized interventions were compared.

Values are reported as mean ± s.d. for normally distributed values or median (interquartile range) for non-normally distributed values. A two-sample *t*-test on paired differences was used to compare normally distributed variables^24^ and the Mann–Whitney–Wilcoxon rank-sum test for data that are not normally distributed. No allowance was made for multiplicity. Outcomes were calculated using GStat software, version 2.3 (University of Cambridge), and statistical analyses were performed using SPSS, version 27 (IBM Software). All *P* values are two-tailed, and *P* values of less than 0.05 were considered to indicate statistical significance.

### Reporting Summary

Further information on research design is available in the Nature Research Reporting Summary linked to this Article.

## Online content

Any methods, additional references, Nature Research reporting summaries, source data, extended data, supplementary information, acknowledgements, peer review information; details of author contributions and competing interests; and statements of data and code availability are available at 10.1038/s41591-021-01453-z.

## Supplementary information

Supplementary Information Supplementary Tables 1–4.

Reporting Summary

Supplementary Note Study protocol.

## Data Availability

The data that support the findings of this study are available from the corresponding author for the purposes of advancing the management and treatment of diabetes. All data shared will be de-identified. The study protocol is available with this paper.

## Code availability

The control algorithm cannot be made publicly available because it is proprietary intellectual property. The control algorithm cannot be used in routine practice in the outpatient setting as regulatory approval has not yet been granted.
