# Publisher Correction: Fully automated closed-loop glucose control compared with standard insulin therapy in adults with type 2 diabetes requiring dialysis: an open-label, randomized crossover trial

**DOI:** 10.1038/s41591-021-01504-5

**Published:** 2021-09-16

**Authors:** Charlotte K. Boughton, Afroditi Tripyla, Sara Hartnell, Aideen Daly, David Herzig, Malgorzata E. Wilinska, Cecilia Czerlau, Andrew Fry, Lia Bally, Roman Hovorka

**Affiliations:** 1 Wellcome Trust – MRC Institute of Metabolic Science, Addenbrooke’s Hospital, Cambridge, UK; 2 Cambridge University Hospitals NHS Foundation Trust, Wolfson Diabetes and Endocrine Clinic, Cambridge, UK; 3 Department of Diabetes, Endocrinology, Nutritional Medicine and Metabolism, Inselspital, Bern University Hospital and University of Bern, Bern, Switzerland; 4 Department of Nephrology and Hypertension, Inselspital, Bern University Hospital and University of Bern, Bern, Switzerland; 5 Department of Renal Medicine, Cambridge University Hospitals NHS Foundation Trust, University of Cambridge, Cambridge, UK

**Keywords:** Type 2 diabetes, End-stage renal disease

Correction to: *Nature Medicine* 10.1038/s41591-021-01453-z, published online 4 August 2021.

In the version of this article initially published, Andrew Fry was incorrectly listed as an equally contributing author. The footnote should read ‘These authors contributed equally: Lia Bally, Roman Hovorka’. The error has been corrected in the HTML and PDF versions of the article.

