# Supplementary material for: Fully automated closed-loop glucose control compared with standard insulin therapy in adults with type 2 diabetes requiring dialysis: an open-label, randomized crossover trial
Source: Nat Med. 2021 Aug 4;27(8):1471–6. doi: 10.1038/s41591-021-01453-z (PMC8363503; doi:10.1038/s41591-021-01453-z)
Supplement: Supplementary file 1 — Supplementary Tables 1–4. [file 41591_2021_1453_MOESM1_ESM.pdf]

nature portfolio

https://doi.org/10.1038/s41591-021-01453-z

Supplementary information

Fully automated closed-loop glucose control compared with standard insulin therapy in adults with type 2 diabetes requiring dialysis: an open-label, randomized crossover trial

In the format provided by the authors and unedited

Supplementary Tables

Table 1 Diabetes treatment regimen at recruitment ` Page 2

Table 2 Comparison of day 1-7 and day 8-20 outcomes Page 3

Table 3 Questionnaires Page 4

Table 4 Closed-loop experience questionnaire Page 5

Table 1. Diabetes treatment regimen at recruitment

	**Overall (n=27)**
Insulin regimen, n (%)	
Basal insulin only	4 (15)
Bolus insulin only	0 (0)
Basal bolus insulin	16 (59)
Pre-mixed insulin	7 (26)
Oral hypoglycaemic agents, n (%)	
DPP-IV inhibitors	3 (11)

DPP-IV - Dipeptidyl peptidase-4

Table 2. Comparison of day 1-7 and day 8-20 outcomes during closed-loop and control therapies.

	Day 1-7		Day 8-20	
	**Closed-loop (n=26)**	**Control (n=26)**	**Closed-loop (n=26)**	**Control (n=26)**
Time spent at glucose levels (%)				
5.6 to 10.0 mmol/L	47.6 (16.1)	37.5 (22.3)	55.8 (12.6)	38.8 (20.7)
3.9 to 10.0 mmol/L	52.6 (18.7)	42.3 (26.7)	59.7 (13.9)	43.6 (24.9)
>10.0 mmol/L	47.0 (18.8)	56.9 (27.4)	40.1 (13.9)	55.7 (25.3)
< 3.9 mmol/L	0.11 (0.00, 0.45)	0.08 (0.00, 0.97)	0.08 (0.00, 0.29)	0.06 (0.00, 0.54)
Mean glucose (mmol/L)	10.5 (1.7)	11.5 (2.7)	9.9 (1.2)	11.6 (3.1)
Standard deviation of glucose (mmol/L)	3.3 (0.9)	3.3 (0.8)	3.0 (0.6)	3.6 (0.9)
CV of glucose (%)	31.7 (5.7)	29.8 (5.8)	30.6 (4.7)	31.2 (5.5)
Total daily insulin dose (units/kg)	0.35 (0.14, 0.56)	0.36 (0.16, 0.59)	0.30 (0.15, 0.54)	0.36 (0.18, 0.58)

Glucose outcomes are based on sensor glucose measurements
Data presented as mean (SD), or median (interquartile range)
CV - coefficient of variation

Table 3. Questionnaire scores.

	Closed-loop	Control	P-value
Hypoglycaemia Fear Survey-II Worry Scale (n=10)	15.0 (5.0, 25.5)	16.5 (6.8, 27.0)	0.153
Hypoglycaemia Confidence Scale (n=22)	3.8 (3.1, 4.0)	3.5 (2.8, 4.0)	0.013
Problem Areas In Diabetes (n=23)	10.0 (1.3, 23.1)	7.5 (1.3, 25.9)	0.868

Data are median (interquartile range)
Hypoglycaemia Fear Survey-II Worry Scale was used only in Cambridge site (n=15)
A Mann–Whitney–Wilcoxon rank-sum test was used to compare the data. No allowance was made for multiplicity.

Table 4. Responses to the closed-loop experience questionnaire.

	Strongly agree	Agree	Neutral	Disagree	Strongly disagree
Q1. I was happy to have my glucose levels controlled automatically by the system	22 (92)	2 (8)	0 (0)	0 (0)	0 (0)
Q2. I spent less time to manage my diabetes (glucose testing, adjusting insulin therapy, keeping a diary, data review…)	19 (79)	3 (13)	2 (8)	0 (0)	0 (0)
Q3. I was less worried about my glucose control	20 (83)	1 (4)	3 (13)	0 (0)	0 (0)
Q4. I slept better during the nights	11 (46)	1 (4)	10 (42)	1 (4)	1 (4)
Q5. I would recommend Closed-Loop to others	23 (96)	1 (4)	0 (0)	0 (0)	0 (0)

Q6. What did you like about the closed-loop system?

- More time for myself due to the automatic blood sugar measurement.
- Much easier than pricking fingers and taking insulin.
- You don't have to think about it, it's easy and stress free.
- Didn't really have to think about diabetes. I didn't have to work out how much insulin to give myself and make myself hypo by mistake. I have lots of health issues to contend with and it was a huge weight off my mind. I felt safer and much more confident.
- Not having to stab my finger several times a day.
- No needles.
- Approved the independence of the automatic blood sugar measurement.
- Having to prick myself less for blood sugar measurement.
- Reduced need to sting for blood sugar measurement.
- The automatic blood glucose control because he often forgets to inject insulin.
- Less time consuming.
- Better blood sugar values thanks to the closed-loop system.
- Simple handling of the devices.
- Very satisfied with the devices. Did not feel disturbed by the devices and was happy about the automatic blood sugar control.
- Approved the automatic blood sugar control which gave him more free time. Did not experience the pump as disturbing and noted that the system caused less scarring or staining than conventional therapies.
- Liked the automatic blood sugar control.
- Satisfied about the automatic blood sugar control.
- No longer had to prick himself so often.
- I felt in control for a change.
- Reassurance and felt more safe.
- Not having to worry about doing anything and remembering to take injection everywhere and injecting in front of people.
- Easy to follow and interesting to see my glucose and insulin levels at a glance at any time.
- Freedom from constant injections and fingertip pricks, peace of mind. In fact everything about the closed-loop system works really well for my diabetes management.
- Everything done for you.
- Not having to worry about injecting.

Q7. What are the things you did not like about the system?

- Having to change from belt clip to neck pouch at night and kept catching my skin.
- Nothing.
- Wires can get in the way; an easier way to scroll through data.
- It took me a while to remember to take the phone everywhere I go.
- Nothing to dislike.
- None.
- None.
- Nothing to dislike about this system.
- Disturbed by the pocket and the amount of material that is needed for closed-loop system.
- During the first night I was slightly concerned about damaging the devices.
- The catheter was the most disturbing component of the closed-loop system in daily activities.
- None.
- Nothing.
- I found the pump very uncomfortable to wear both day and night.
- The amount of equipment that is needed to manage the diabetes.
- In the beginning of the interventional period, the patient had some technical problems with the smartphone.
- Being disturbed by the pocket of the pump.
- The pocket worn around the neck.
- The amount of material that is needed for the closed-loop system.
- The plasters that were used to secure the catheter and the sensor, especially while taking a shower.
- Slightly concerned about the pocket, because it was not 100% waterproof.
- Bigger insulin reservoir capacity.

Q8. Would you like the closed-loop system to have additional features? If yes, which ones?

- Simplification of the closed-loop devices.
- A model with no need to change the catheter every other day or without any catheter.
- All three main components united in one single device.
- Bigger insulin reservoir.
- Wanted the pump at another part of the body or in trouser pocket.
- Personal smartphone for the use of the CL system instead of a study phone.
- Add pictures to meal plan.
