# Supplementary material for: Fully automated closed-loop glucose control compared with standard insulin therapy in adults with type 2 diabetes requiring dialysis: an open-label, randomized crossover trial
Source: Nat Med. 2021 Aug 4;27(8):1471–6. doi: 10.1038/s41591-021-01453-z (PMC8363503; doi:10.1038/s41591-021-01453-z)
Supplement: Supplementary file 2 — Reporting Summary [file 41591_2021_1453_MOESM2_ESM.pdf]

nature research

Corresponding author(s): Dr Charlotte Boughton

Last updated by author(s): Jun 25, 2021

Reporting Summary

Nature Research wishes to improve the reproducibility of the work that we publish. This form provides structure for consistency and transparency in reporting. For further information on Nature Research policies, see our Editorial Policies and the Editorial Policy Checklist.

Statistics

For all statistical analyses, confirm that the following items are present in the figure legend, table legend, main text, or Methods section.

n/a	Confirmed	
☐	☒	The exact sample size (*n*) for each experimental group/condition, given as a discrete number and unit of measurement
☐	☒	A statement on whether measurements were taken from distinct samples or whether the same sample was measured repeatedly
☐	☒	The statistical test(s) used AND whether they are one- or two-sided<br>*Only common tests should be described solely by name; describe more complex techniques in the Methods section.*
☒	☐	A description of all covariates tested
☐	☒	A description of any assumptions or corrections, such as tests of normality and adjustment for multiple comparisons
☐	☒	A full description of the statistical parameters including central tendency (e.g. means) or other basic estimates (e.g. regression coefficient) AND variation (e.g. standard deviation) or associated estimates of uncertainty (e.g. confidence intervals)
☐	☒	For null hypothesis testing, the test statistic (e.g. *F*, *t*, *r*) with confidence intervals, effect sizes, degrees of freedom and *P* value noted<br>*Give P values as exact values whenever suitable.*
☒	☐	For Bayesian analysis, information on the choice of priors and Markov chain Monte Carlo settings
☒	☐	For hierarchical and complex designs, identification of the appropriate level for tests and full reporting of outcomes
☒	☐	Estimates of effect sizes (e.g. Cohen's *d*, Pearson's *r*), indicating how they were calculated

Our web collection on statistics for biologists contains articles on many of the points above.

Software and code

Policy information about availability of computer code

Data collection	The CamAPS HX closed-loop app (CamDiab, Cambridge, UK) resides on an unlocked Android phone, receives sensor glucose data from a Dexcom G6 transmitter (Dexcom, San Diego, CA, USA), and uses Cambridge adaptive model predictive control algorithm (version 0.3.71) to direct insulin delivery on a Dana Diabecare RS pump (Diabecare, Sooil, South Korea). Sensor glucose and insulin data were obtained from the Diasend/Glooko (https://diasend.com//en) data management platform.
Data analysis	Outcomes were calculated using GStat software, version 2.3 (University of Cambridge), and statistical analyses were performed using SPSS, version 27 (IBM Software, Hampshire, UK).

For manuscripts utilizing custom algorithms or software that are central to the research but not yet described in published literature, software must be made available to editors and reviewers. We strongly encourage code deposition in a community repository (e.g. GitHub). See the Nature Research guidelines for submitting code & software for further information.

Data

Policy information about availability of data

All manuscripts must include a data availability statement. This statement should provide the following information, where applicable:

- Accession codes, unique identifiers, or web links for publicly available datasets
- A list of figures that have associated raw data
- A description of any restrictions on data availability

The data that support the findings of this study are available from the corresponding author for the purposes of advancing management and treatment of diabetes. All data shared will be de-identified. Study protocol is available with publication.

Field-specific reporting

Please select the one below that is the best fit for your research. If you are not sure, read the appropriate sections before making your selection.

☒ Life sciences ☐ Behavioural & social sciences ☐ Ecological, evolutionary & environmental sciences

For a reference copy of the document with all sections, see nature.com/documents/nr-reporting-summary-flat.pdf

Life sciences study design

All studies must disclose on these points even when the disclosure is negative.

Sample size	This was an exploratory study aiming for 32 subjects with at least 48 hours of data. Since previous studies using closed-loop in an inpatient setting may not provide reliable information about the standard deviation of the primary endpoint in this particular population (outpatients receiving maintenance dialysis), no formal power calculation was applied. The sample size corresponds to the sample size of previous feasibility closed-loop randomised trials (9,11).
Data exclusions	All analyses were carried out on an intention-to-treat basis. We analysed end points from participants with at least 48 h of sensor glucose data in both study periods. Of 27 randomized participants, one participant was withdrawn from the study post-randomisation as they required hospital admission and died before the start of the first intervention period (control). Two participants stopped a study period early; one during the second period (control) due to bereavement and one during the first period (closed-loop) due to local covid-19 restrictions. These participants both completed a minimum of 48 hours in both study periods and were included in the analysis.
Replication	The current manuscript reports the results of a clinical trial. The methods have been described in detail s in the text and study protocol to allow replication
Randomization	Eligible participants were randomly assigned to either initial use of fully closed-loop glucose control with faster acting insulin aspart for 20 days followed by standard multiple daily insulin injection therapy for 20 days or vice versa. Randomisation was done using a computer-generated sequence with a permuted block design (block size 4) and stratified by centre.
Blinding	Participants and investigators were not masked to the intervention being used during each period due to the nature of the interventions precluding the ability to mask.

Reporting for specific materials, systems and methods

We require information from authors about some types of materials, experimental systems and methods used in many studies. Here, indicate whether each material, system or method listed is relevant to your study. If you are not sure if a list item applies to your research, read the appropriate section before selecting a response.

Materials & experimental systems

n/a	Involved in the study	
☒	☐	Antibodies
☒	☐	Eukaryotic cell lines
☒	☐	Palaeontology and archaeology
☒	☐	Animals and other organisms
☐	☒	Human research participants
☐	☒	Clinical data
☒	☐	Dual use research of concern

Methods

n/a	Involved in the study	
☒	☐	ChIP-seq
☒	☐	Flow cytometry
☒	☐	MRI-based neuroimaging

Human research participants

Policy information about studies involving human research participants

Population characteristics	From 21 October 2019 to 3 November 2020, 27 participants were enrolled and randomised (17 men, 9 women, average age 68±11 years [mean ±SD] and average diabetes duration 20±10 years).
Recruitment	Participants were recruited from dialysis centres and nephrology and diabetes outpatient clinics at Addenbrooke's Hospital in Cambridge, United Kingdom, and Inselspital, University Hospital of Bern, Switzerland. Inclusion criteria included age 18 years and older, type 2 diabetes requiring subcutaneous insulin therapy and end stage renal failure requiring maintenance dialysis (haemodialysis or peritoneal dialysis). Exclusion criteria included type 1 diabetes, pregnancy or breast-feeding, severe visual or hearing impairment and any physical or psychological disease or the use of medication(s) likely to interfere with the conduct of the trial or interpretation of the results.<br>Recruitment rate was approximately 50% of those who were approached and eligible to participate in the study and included only one participant using peritoneal dialysis, which may limit generalisablity of the findings.
Ethics oversight	The study protocol was approved by the local Research Ethics Committees (London – Stanmore Ethics Committee, UK, and

Ethics oversight | Ethics Committee Bern, Switzerland) and Regulatory Authorities (MHRA and Swissmedic). The safety aspects of the trial were overseen by an independent Data and Safety Monitoring Board.

Note that full information on the approval of the study protocol must also be provided in the manuscript.

Clinical data

Policy information about clinical studies

All manuscripts should comply with the ICMJE guidelines for publication of clinical research and a completed CONSORT checklist must be included with all submissions.

Clinical trial registration

The study was registered with ClinicalTrials.gov NCT04025775.

Study protocol

Study protocol is available with publication

Data collection

Participants were enrolled and randomised from 21 October 2019 to 3 November 2020 at Addenbrooke's Hospital in Cambridge, United Kingdom, and Inselspital, University Hospital of Bern, Switzerland. Participant demographics and medical history, body weight and height, glycated haemoglobin (HbA1c) and total daily insulin dose were recorded at enrolment. Study visits were undertaken at the hospital or local dialysis centre.

Participants were randomly assigned to either initial use of fully closed-loop glucose control with faster acting insulin aspart for 20 days followed by standard multiple daily insulin injection therapy for 20 days or vice versa. Glycaemic and insulin endpoints were collected from each 20-day study period. Sensor glucose and insulin data are automatically uploaded to the Diasend/Glooko data management platform. Body weight pre- and post-dialysis was recorded at each dialysis session (or daily if on peritoneal dialysis) during each 20-day study period. Psychosocial assessments were measured using questionnaires collected at the end of each study period.

Outcomes

The primary endpoint was the percentage of time the sensor glucose measurement was in the target glucose range of 5.6 to 10.0 mmol/L during the 20-day study period. This target glucose range was selected in line with recommendations for less stringent glucose control in this population due to their high risk for hypoglycaemia and related adverse events (5, 6, 16-18).

Other key endpoints are the percentage of time spent with sensor glucose above 10.0 mmol/L, mean sensor glucose, and the percentage of time spent with sensor glucose below 3.9 mmol/L. Secondary efficacy endpoints included time spent with sensor glucose below 5.6 mmol/L and below 3.0 mmol/L, time spent with sensor glucose levels in significant hyperglycaemia (>20mmol/L) and the total daily insulin dose. Glucose variability was evaluated by the standard deviation and the coefficient of variation of sensor glucose utilising data collected from the whole study period. The between-day coefficient of variation of sensor glucose was calculated from daily mean glucose values (0000-2359).

Safety end-points included severe hypoglycaemia (capillary glucose <2.2 mmol/L, or requiring assistance of another person), along with other adverse events and serious adverse events and device deficiencies.

Exploratory analyses included a subset of glucose and insulin metrics during the first 7 days and during the subsequent period of day 8 to day 20 (time in target, time above target, time in hypoglycaemia (<3.9 mmol/L), mean sensor glucose, standard deviation and the coefficient of variation of sensor glucose, and total daily insulin dose) to limit the number of comparisons. Variability of glucose and insulin requirements between dialysis and non-dialysis days was assessed using coefficient of variation of sensor glucose and insulin requirements between dialysis days (0000-2359) and non-dialysis days (0000-2359). Mean inter-dialytic weight gain was calculated for each study period.

Psychosocial assessments were measured using questionnaires collected at the end of each study period and closed-loop participants' experience at the end of the closed-loop period.
